# Supplementary material for: Rapid retreat of Berry Glacier, West Antarctica, linked to seawater intrusions revealed by radar interferometry
Source: Nat Commun. 2025 Oct 21;16:9292. doi: 10.1038/s41467-025-64330-0 (PMC12540669; doi:10.1038/s41467-025-64330-0)
Supplement: Supplementary file 1 — Supplementary Information [file 41467_2025_64330_MOESM1_ESM.pdf]

Supplementary Figures

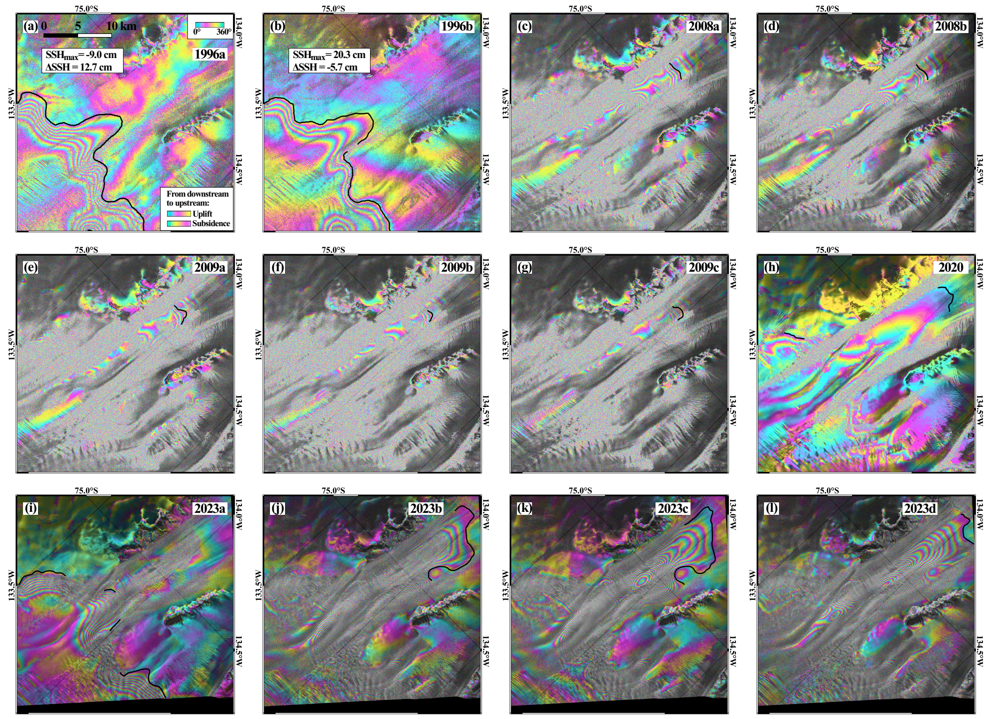

Supplementary Fig. 1 Differential interferograms from ERS-1/2, ALOS-1/2 PALSAR and RCM. (a-b) ERS-1/2; (c-g) ALOS-1 PALSAR; (h) ALOS-2 PALSAR; (i-l) RCM. Black lines are GL positions. Orbit pairs and acquisition times used in the interferograms are listed in the table below in the same respective order.

Data	Orbit Pairs	Dates
1996a ERS-1/2	23886/4213, 24387/4714	96-02-08/96-02-09, 96-03-14/96-03-15
1996b ERS-1/2	23929/4256, 24430/4757	96-02-11/96-02-12, 96-03-17/96-03-18
2008a ALOS-1	14242/14913, 14913/15584	08-09-26/08-11-11, 08-11-11/08-12-27
2008b ALOS-1	14417/15088, 15088/15759	08-10-08/08-11-23, 08-11-23/09-01-08
2009a ALOS-1	19435/20106, 20106/20777	09-09-17/09-11-02, 09-11-02/09-12-18
2009b ALOS-1	19610/20281, 20281/20952	09-09-29/09-11-14, 09-11-14/09-12-30
2009c ALOS-1	19785/20456, 20456/21127	09-10-11/09-11-26, 09-11-26/10-01-11
2020 ALOS-2	34456/34663, 34663/34870	20-10-09/20-10-23, 20-10-23/20-11-06
2023a RCM	19404/19464, 19643/19703	23-01-03/23-01-07, 23-01-19/23-01-23
2023b RCM	19404/19464, 19703/19762	23-01-03/23-01-07, 23-01-23/23-01-27
2023c RCM	19643/19703, 19703/19762	23-01-19/23-01-23, 23-01-23/23-01-27
2023d RCM	19643/19762, 19762/19882	23-01-19/23-01-27, 23-01-27/23-02-04

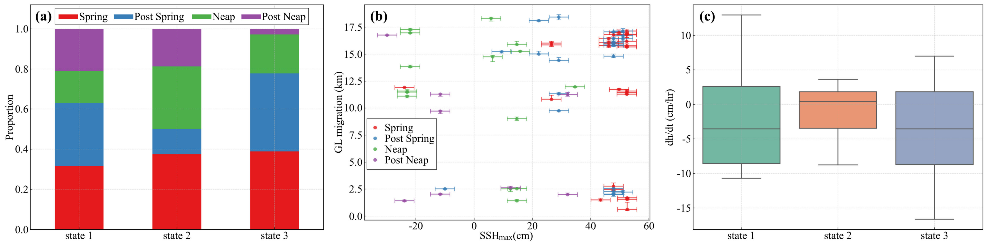

Supplementary Fig. 2 Tidal phase effects on grounding line migration. (a) Tidal phase distribution of maximum tide height among 3 or 4 acquisition times for each GL state; (b) GL migration vs. maximum SSH by tidal phase; (c) distribution of dh/dt at maximum tide height by GL state.

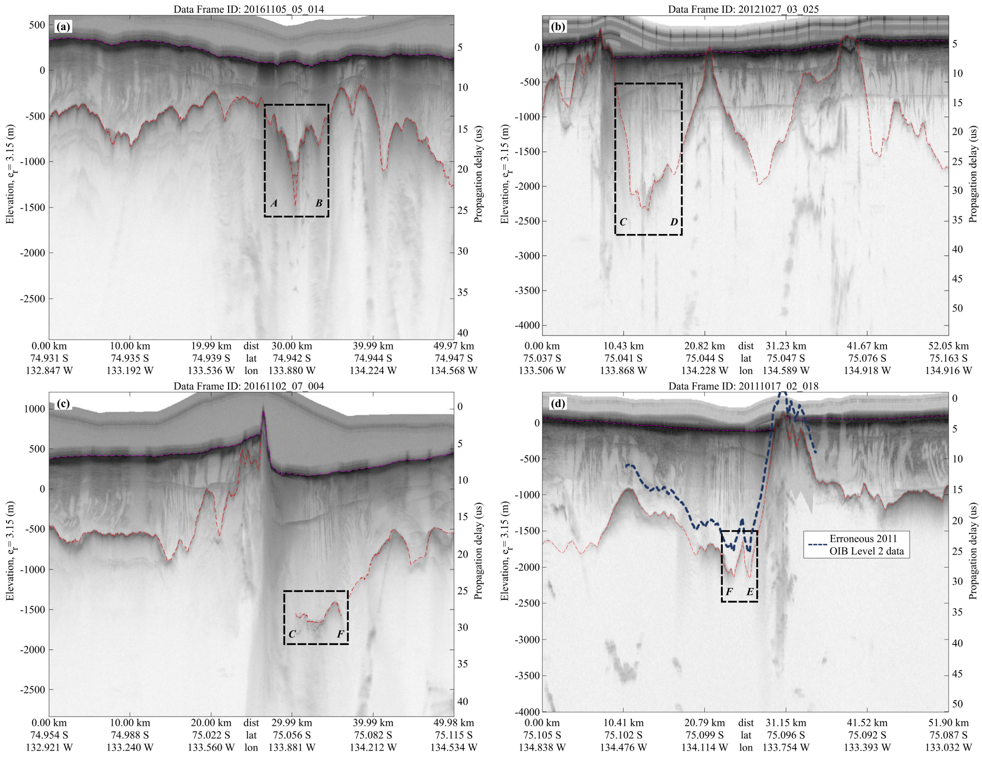

Supplementary Fig. 3 Echogram from Multichannel Coherent Radar Depth Sounder (MCoRDS). Along profiles (a) A-B from Nov 5, 2016, (b) C-D from Oct 27, 2012, (c) C-F from Nov 2, 2016, and (d) F-E from Oct 17, 2011. Picked ice surface is purple dash. Bottom pick is red dash. Black box shows the domain in Supplementary Fig. 4. The direction of profile F-E in (d) is west-east, which is opposite to that in Supplementary Fig. 4d. Original MCoRDS data from level 2 data (dark blue dash) in (d) have a 300-m offset due to an error in platform elevation.

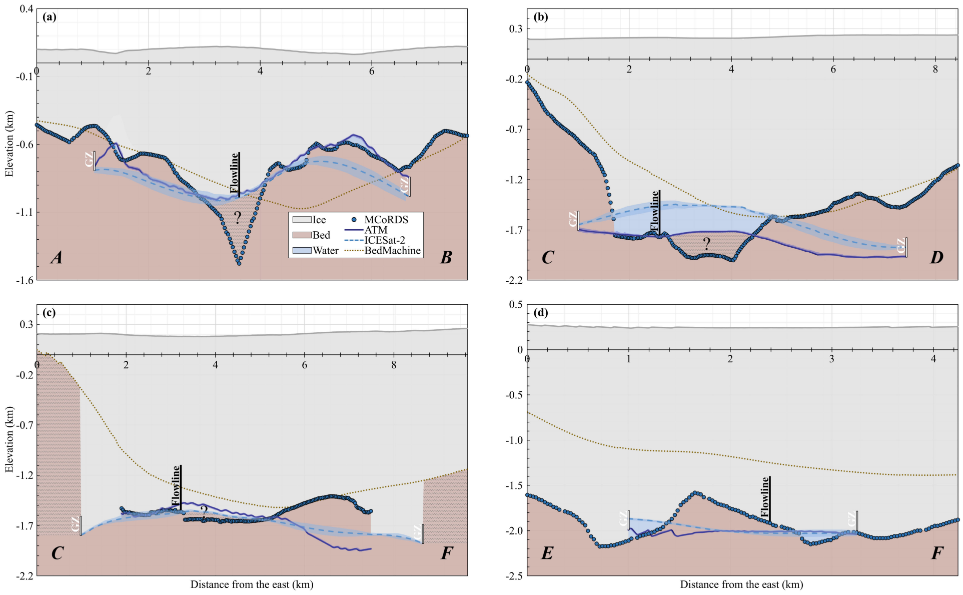

Supplementary Fig. 4 Ice elevation and bed elevation along OIB tracks. (a) A-B, (b) C-D, (c) C-F, and (d) E-F. Surface elevation (grey line) from Airborne Topographic Mapper (ATM), ice draft (blue line) deduced from ATM, and b (brown dotted) from BMv3.7. Blue dash is flotation limit from ICESat-2 on Jan 1, 2020; blue dots are ice bottoms from MCoRDS. Blue shaded areas are the uncertainty of the derived ice drafts. Bed layers marked with question marks are due to questionable picked ice bottoms based on echo strength profiles (Supplementary Fig. 3) because the bed from MCoRDS cannot be lower than the beds from flotation. Acquisition times of OIB containing ATM and MCoRDS are respectively from Nov 5, 2016 (a), Oct 27, 2012 (b), Nov 2, 2016 (c) and Oct 17, 2011 (d).

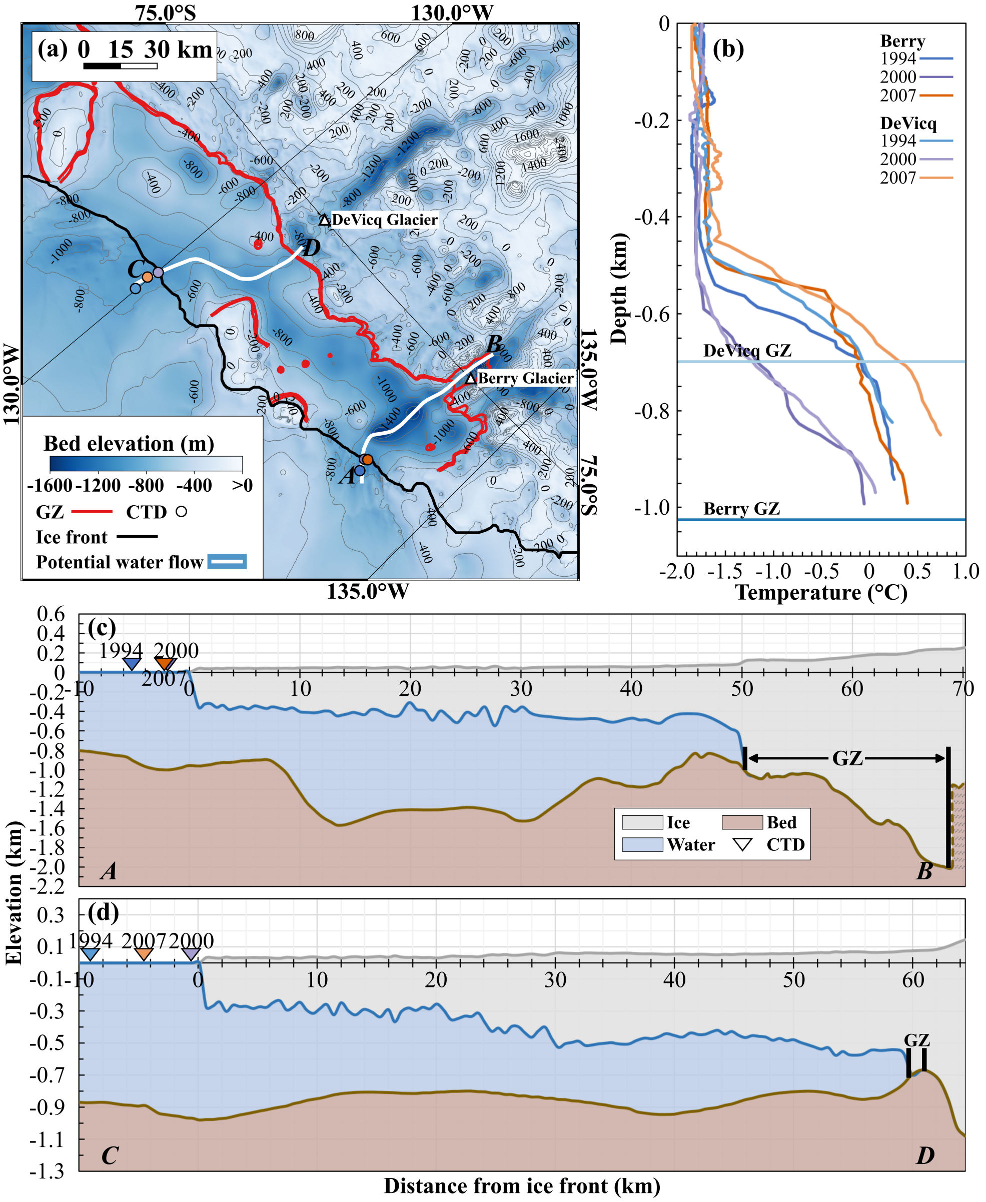

Supplementary Fig. 5 Bed topography, *b*, in the west Getz Ice Shelf. (a) *b* in West Getz Ice Shelf from BMv3.7 color coded from -1600 m (blue) to 0 m (white), with IGZ (red) from year 2018, CTD at ice front (colored dots) from Feb 22, 1994 (19940222_2240_WOD09CTDUS_3596), Feb 26, 2000 (20000226_0950_WOD09CTDUS_3596) and Mar 6, 2007 (20070306_0325_WOD09CTDUS_3596) for Berry, and Feb 23, 1994 (19940223_0755_WOD09CTDUS_3596), Feb 27, 2000 (20000227_0025_WOD09CTDUS_3596) and Mar 4, 2007 (20070304_1831_WOD09CTDUS_3596) for DeVicq (Jacobs et al. 2013), and 200-m contour level in bed topography. Potential water path from the ice front to the IGZ of Berry and DeVicq is white; (b) temperature as depth profiles from 1994 to 2007, from the CTD measurements made at sampling sites in (a). The blue line indicates the entrance depth of the IGZ; surface elevation, ice bottom and *b* from BMv3.7 along profiles A-B (c) and C-D (d) in (a). The locations of the CTD measurements are marked in front of the ice shelf.

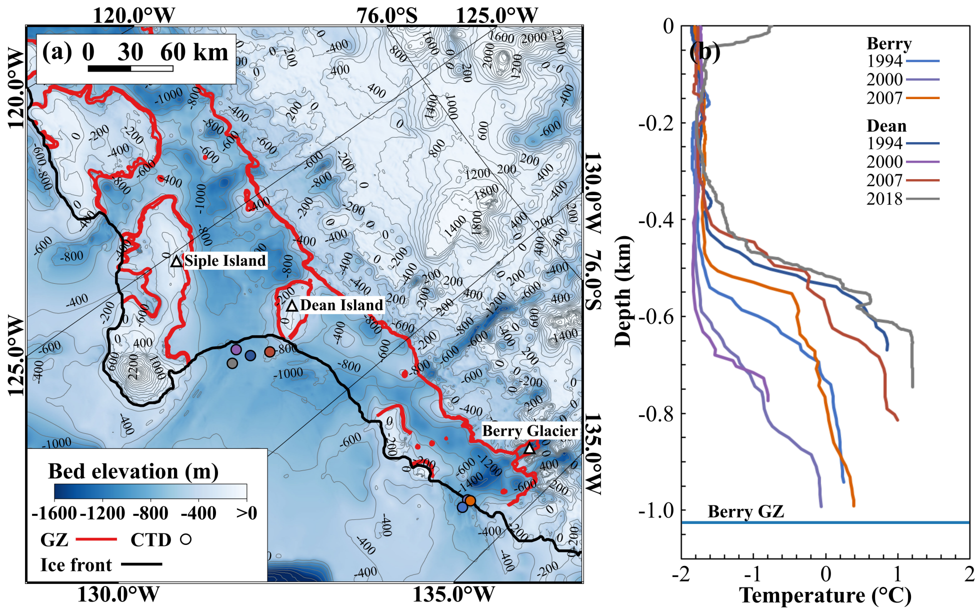

Supplementary Fig. 6 Ocean temperature from 1994 to 2018 in the west Getz Ice Shelf. (a) *b* in West Getz Ice Shelf from BMv3.7 color coded from -1600 m (blue) to 0 m (white), with IGZ (red) from year 2018, CTD at ice front (colored dots) from Feb 22, 1994 (19940222_2240_WOD09CTDUS_3596), Feb 26, 2000 (20000226_0950_WOD09CTDUS_3596) and Mar 6, 2007 (20070306_0325_WOD09CTDUS_3596) for Berry, and Feb 23, 1994 (19940223_1440_WOD09CTDUS_3596), Feb 27, 2000 (20000227_1130_WOD09CTDUS_3596), Mar 4, 2007 (20070304_1120_WOD09CTDUS_3596) and Jan 19, 2018 (ANA08B18CTD1) for Dean, and 200-m contour level in bed topography. The CTD from year 2018 located in front of Dean Island is the latest data in the west Getz Ice Shelf; (b) temperature as depth profiles from 1994 to 2018, from the CTD measurements made at sampling sites in (a).

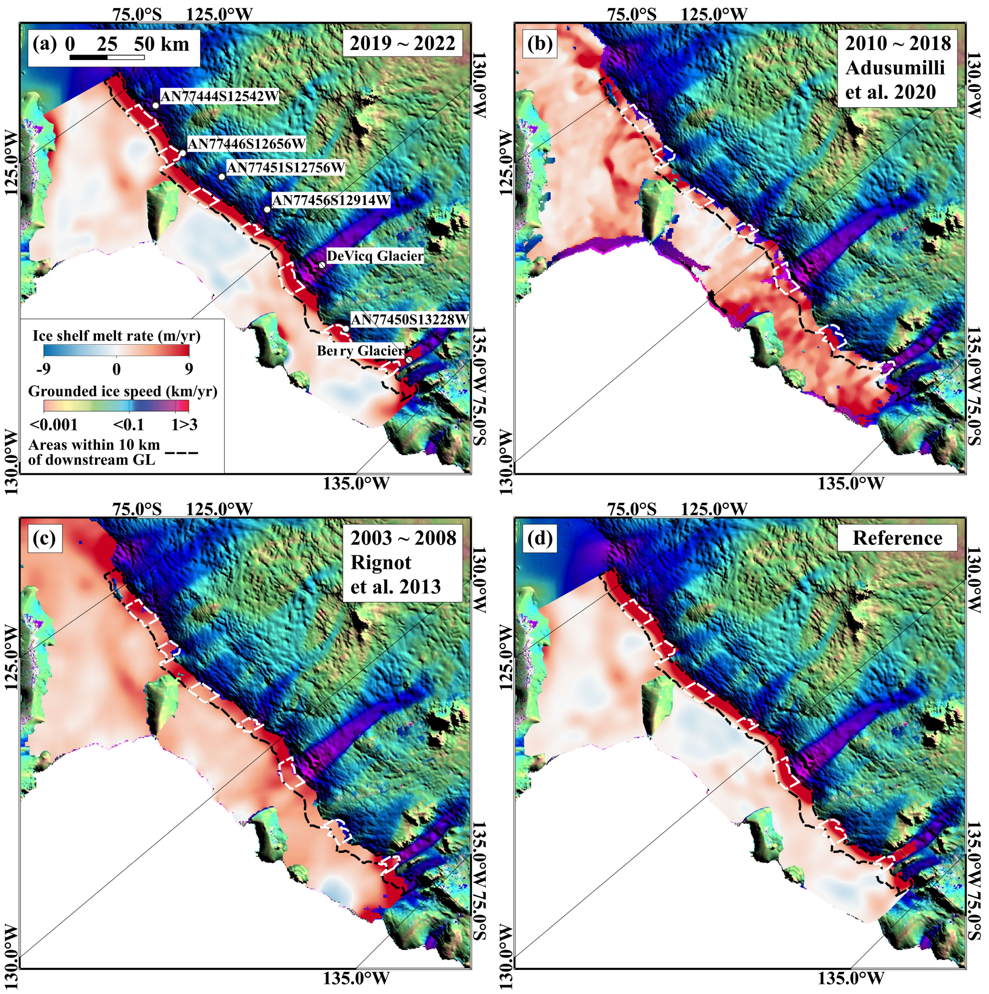

Supplementary Fig. 7 Basal melt rate, $\dot{b}$, on West Getz Ice Shelf. Calculated for (a) 2019-2022 (this study), (b) 2010-2018 from Adusumilli et al. 2020, and (c) 2003-2008 from Rignot et al. 2013, overlaid on ice speed and glacier names; (d) reference melt rate calculated with 2007-2008 ice velocity, reference SMB (1979 to 2004), and 2007-2008 ice thickness assuming steady state or no thinning. Black dash line in (a-d) delineates area within 10 km downstream of IGZ. The average melt rate within the 10-km wide region (white dash line) for each glacier is shown in the table below, in meters per year.

Places	2019-2022	2010-2018	2003-2008	Reference
Entire 10-km region	8.1	5.8	7.0	5.1
Berry Glacier	14.9	Null	9.1	7.1
AN77450S13228W	6.8	9.2	4.2	4.5
DeVicq Glacier	8.6	7.7	6.9	5.4
AN77456S12914W	6.0	5.6	6.1	5.1
AN77451S12756W	5.3	4.9	4.7	5.3
AN77446S12656W	7.4	6.7	6.4	5.4
AN77444S12542W	6.9	4.3	7.8	6.3

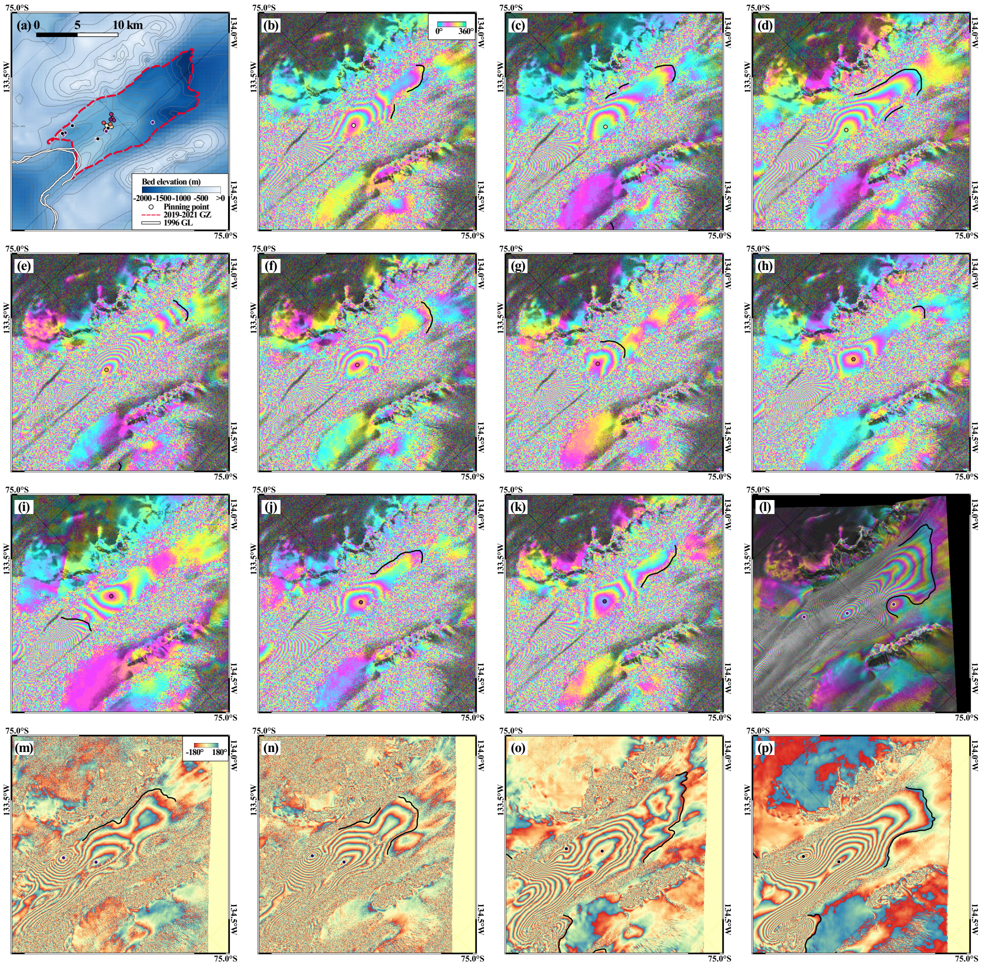

Supplementary Fig. 8 Pinning points in the ice grounding zone (IGZ) of Berry Glacier. (a) Dots mark pinning points based on double-difference interferograms (b-p) in 2019-2021 IGZ overlaid on bed topography combining BMv3.7 (outside IGZ) and ice base from flotation using a WorldView DEM (inside IGZ) color coded from -2000 m (blue) to 0 m (white); GL positions (black line) and pinning points (dot) derived from S1 (b-k), RCM (l) and CSK (m-p). The color of dots in (b-p) respectively correspond to the colors of the points in (a).
