# Supplementary material for: Rapid retreat of Berry Glacier, West Antarctica, linked to seawater intrusions revealed by radar interferometry
Source: Nat Commun. 2025 Oct 21;16:9292. doi: 10.1038/s41467-025-64330-0 (PMC12540669; doi:10.1038/s41467-025-64330-0)
Supplement: Supplementary file 2 — Description of Additional Supplementary Files [file 41467_2025_64330_MOESM2_ESM.pdf]

Description of Additional Supplementary Files

File name: Supplementary Video 1
Description: Animation of a time series of Landsat 7/8 panchromatic band images with 1996 GL and 2019-2021 IGZ covering Berry Glacier, West Antarctica, from 2000-2022 (gaps in 2004-2012).
